# When Clinical Improvement Does Not Reflect Radiological Response: A Culture-Negative Giant Pyogenic Liver Abscess

**DOI:** 10.3390/healthcare14101262

**Published:** 2026-05-07

**Authors:** Zuzanna Żak-Skryśkiewicz, Justyna Nowak, Tomasz Oleksiuk, Przemysław Witek

**Affiliations:** 1 Department of Internal Medicine, Endocrinology and Diabetes, Medical University of Warsaw, 02-091 Warsaw, Poland; przemyslaw.witek@wum.edu.pl; 2 Department of Internal Medicine, Endocrinology and Diabetes, Mazovian Bródnowski Hospital, Kondratowicza 8, 03-242 Warsaw, Poland; j.zielinska2wl@gmail.com; 3 Department of Radiology, Mazovian Bródnowski Hospital, Kondratowicza 8, 03-242 Warsaw, Poland; olekstom@yahoo.com

**Keywords:** pyogenic liver abscess, drainage, computed tomography, diabetes mellitus, bacterial infections

## Abstract

**Background:** Management of giant pyogenic liver abscesses (PLA) remains challenging, particularly in culture-negative cases, where clinical improvement may not reflect adequate local disease control. **Case Description:** A 65-year-old woman with well-controlled type 2 diabetes mellitus presented with several weeks of systemic symptoms, marked inflammatory response, cholestatic liver injury, and acute kidney dysfunction. Contrast-enhanced computed tomography (CT) and magnetic resonance imaging (MRI) revealed a large, multiloculated hepatic lesion measuring approximately 10 cm, consistent with a giant PLA. Empirical broad-spectrum antimicrobial therapy resulted in rapid clinical and biochemical improvement; however, follow-up imaging demonstrated further enlargement of the abscess. Microbiological cultures from blood, urine, and the abscess cavity remained negative. In view of radiological progression, CT-guided percutaneous catheter drainage was performed, resulting in effective evacuation and subsequent lesion regression. Long-term follow-up confirmed complete resolution without recurrence. **Conclusions:** This case highlights that clinical and laboratory improvement alone may be insufficient to assess treatment response in giant, culture-negative PLA. Serial imaging plays a key role in identifying inadequate local disease control and guiding timely escalation to image-guided intervention.

## 1. Introduction

Pyogenic liver abscess (PLA) is a rare but potentially life-threatening condition, with an annual incidence in Europe and North America estimated at 1–15 cases per 100,000 persons [1]. Marked geographic variation in pathogen distribution has been observed; in Asian populations, *Klebsiella pneumoniae* predominates (69–86% of cases) [2,3], whereas in Western countries, *Escherichia coli* and *Streptococcus anginosus* are more frequently isolated [4,5,6].

Despite advances in imaging and antimicrobial therapy, PLA remains associated with a substantial morbidity and mortality of approximately 4–8% [2,4,7]. Well-established risk factors include hepatobiliary conditions such as liver cirrhosis, hepatobiliary malignancies, and biliary tract obstruction, as well as intestinal diseases, diabetes mellitus (DM), liver transplantation, and immunocompromised states [8].

Microbiological identification is a key component of diagnosis, yet blood cultures are positive in only 25–43% of cases, while abscess aspirates yield pathogens in 78–91% [4,7,9]. Nevertheless, culture-negative PLA (CNPLA) remains common, reported in 31–38% of patients [7,10]. CNPLA does not exclude severe infection, and management often relies on clinical, laboratory, and imaging findings rather than pathogen identification alone [4,7]. In this context, radiological assessment plays an essential role not only in diagnosis but also in the dynamic evaluation of treatment response.

Differential diagnosis of PLA should be considered, particularly in cases with atypical presentation or negative microbiological findings. Imaging features may overlap with primary and secondary hepatic malignancies, as well as benign lesions such as hemangioma or focal nodular hyperplasia [11,12]. Infectious mimickers, including amebic liver abscess and echinococcosis, should also be considered depending on the epidemiological context [11]. Accurate diagnosis requires integration of clinical, laboratory, and radiological data.

Ultrasonography is often the first-line modality due to its availability and safety, whereas contrast-enhanced computed tomography (CT) provides superior sensitivity and allows precise assessment of lesion size, multiloculation, and potential complications [13]. Magnetic resonance imaging (MRI) may offer additional diagnostic clarification in equivocal cases [13]. Emerging techniques such as contrast-enhanced ultrasound have further improved lesion characterization, although their availability remains limited in routine clinical practice [14].

Lesion size represents an important modifier of disease course and management strategy. PLAs have been categorized according to maximal diameter, with “giant” abscesses defined as lesions measuring ≥10 cm [9]. Giant abscesses have been associated with increased disease complexity, prolonged hospitalization, and a higher likelihood of inadequate response to antimicrobial therapy alone, frequently necessitating interventional source control [9]. Despite their association with a more complicated clinical course, current evidence does not demonstrate a size-dependent alteration in microbiological etiology, as the distribution of predominant pathogens appears comparable across different abscess size categories [15].

Management typically involves prolonged antimicrobial therapy combined with image-guided drainage when source control cannot be achieved pharmacologically. While smaller abscesses may respond to antibiotics alone, larger or multiloculated lesions frequently require percutaneous catheter drainage (PCD), with surgical drainage (SD) reserved for selected refractory cases [16].

We report a case of a giant CNPLA with discordant clinical and radiological responses to therapy in an otherwise immunocompetent patient whose only identifiable risk factor was well-controlled type 2 DM.

## 2. Case Presentation

A 65-year-old woman with a history of type 2 DM treated with oral hypoglycemic agents, hypertension, and hypothyroidism on levothyroxine replacement therapy was acutely admitted to the Internal Medicine Department following approximately four weeks of progressive systemic symptoms, including fever, profound weakness, gastrointestinal complaints, unintentional weight loss, confusion, and jaundice. Her most recent glycated hemoglobin (HbA1c) level was 6.2%, consistent with adequate long-term glycemic control. Apart from the comorbidities listed above, the patient had no known chronic inflammatory or malignant diseases and denied excessive alcohol consumption or illicit drug use. Importantly, she had no history of prior abdominal surgery or gastrointestinal procedures.

On admission, the patient appeared acutely ill, mildly confused yet oriented to person and place, with clinical signs of dehydration and scleral icterus. Her heart rate was 109 beats per minute, blood pressure measured 114/68 mmHg, and oxygen saturation was 88–89% while breathing ambient air. Lung auscultation revealed symmetric vesicular breath sounds with subtle crackles over the mid-zone of the left lung. Abdominal examination demonstrated a soft, non-tender abdomen without palpable masses or peritoneal signs. No peripheral edema was observed.

Initial laboratory evaluation revealed a marked systemic inflammatory response (C-reactive protein concentration [CRP], 364.7 mg/L; procalcitonin concentration [PCT], 56.08 ng/mL), accompanied by leukocytosis (white blood cell count, 17.63 × 10^9^/L) with neutrophilia (16.27 × 10^9^/L, 92.3%), cholestatic liver injury (aspartate aminotransferase activity [AST], 267 U/L; alanine aminotransferase activity [ALT], 237 U/L; total bilirubin concentration, 2.6 mg/dL; gamma-glutamyl transferase activity [GGT], 84 U/L) and acute renal dysfunction (creatinine concentration, 1.6 mg/dL; estimated glomerular filtration rate, 32 mL/min/1.73 m^2^).

**Figure 1:**
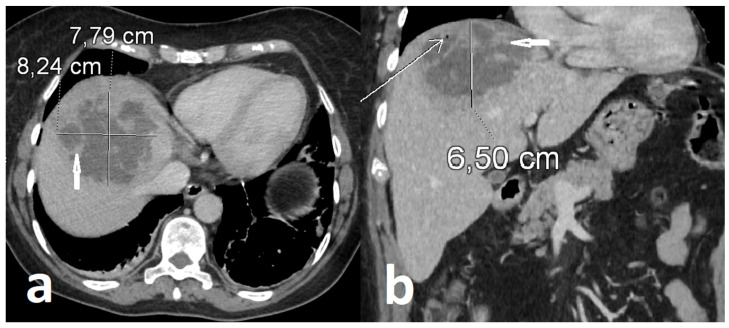
Hepatic abscess in initial contrast-enhanced CT scan measuring approx. 8.2 × 7.8 × 6.5 cm: axial (**a**) and coronal (**b**) plane views. Abscess located in the subdiaphragmatic region, reaching the surface of the liver with focal abutment of the diaphragm, contains irregular internal septa (short arrows) and intralesional gas (long arrow).

Serological screening excluded hepatitis B virus infection (HBsAg, anti-HBc, and anti-HBs), hepatitis C virus infection (anti-HCV), and human immunodeficiency virus infection (HIV Ag/Ab). Tumor markers, including cancer antigen 125 (CA-125), carcinoembryonic antigen (CEA), and alpha-fetoprotein (AFP), were not significantly elevated. Abdominal ultrasonography revealed a large complex lesion in the right hepatic lobe characterized by mixed solid and fluid components with thick internal septations, raising suspicion for abscess formation. Urgent contrast-enhanced abdominal CT demonstrated a heterogeneous hepatic lesion with irregular margins and internal septations, consistent with an advanced infectious process (Figure 1).

**Figure 2:**
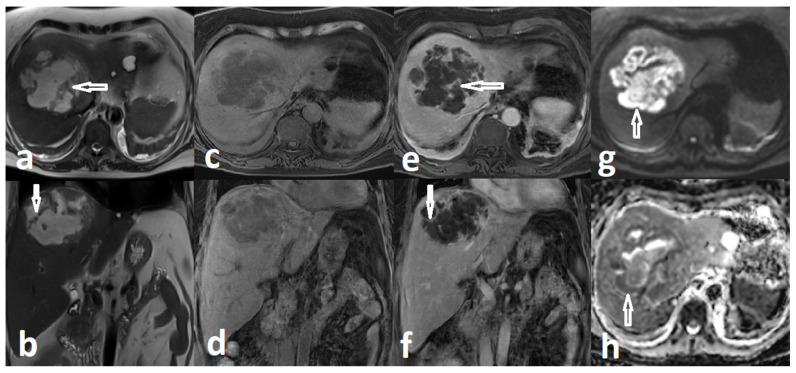
Hepatic abscess in MR scan, from left: T2WI in axial (**a**) and coronal (**b**) plane, T1WI without contrast in axial (**c**) and coronal (**d**) plane, T1WI with contrast in axial (**e**) and coronal (**f**) plane, DWI (**g**) and ADC map (**h**) in axial plane. Coronal images show abscess reaching the surface of the liver with abutment of the diaphragm. Abscess content demonstrates high T2W signal, low T1W signal and inhomogeneous restriction diffusion in DWI/ADC—predominantly high signal in DWI (short arrow in (**g**)) and predominantly low signal, especially peripherally, on ADC map (short arrow in (**h**)). T2W images clearly visualize low signal internal septa (short arrows in (**a**,**b**)). Post contrast T1W images show enhancement of irregular internal septa (short arrows in (**e**,**f**)).

Further diagnostic clarification was achieved with contrast-enhanced MRI. The lesion was located predominantly in hepatic segment VIII, with extension toward segments IV and VII, and measured approximately 95 × 100 × 75 mm. On T2-weighted images, it appeared predominantly hyperintense and heterogeneous with internal septations. Post-contrast T1-weighted sequences demonstrated irregular peripheral and septal enhancement, with adjacent parenchymal hyperenhancement suggestive of an inflammatory reaction. Diffusion-weighted imaging (DWI) revealed marked restricted diffusion within the lesion, confirmed on the apparent diffusion coefficient (ADC) map. Small intralesional gas foci were suspected. The lesion extended to the liver surface in the subdiaphragmatic region, demonstrating focal abutment of the diaphragm and the adjacent pericardium. These findings were consistent with a multiloculated PLA (Figure 2).

**Figure 3:**
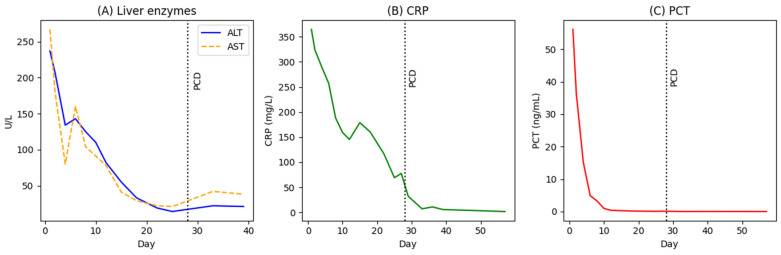
Trends in biochemical parameters during hospitalization. (**A**) Liver enzymes, including alanine aminotransferase (ALT) and aspartate aminotransferase (AST), (**B**) C-reactive protein (CRP), and (**C**) procalcitonin (PCT), demonstrating marked biochemical improvement despite ongoing radiological progression of the hepatic abscess. The vertical line indicates the timing of percutaneous catheter drainage (PCD).

Empirical broad-spectrum antimicrobial therapy with intravenous vancomycin, meropenem, and metronidazole was initiated promptly at reduced doses due to impaired renal function. Following rapid improvement in renal parameters, standard dosing was introduced, including meropenem 1 g every 8 h, vancomycin 1 g every 12 h, and metronidazole 500 mg every 8 h. Blood and urine cultures, obtained prior to the initiation of antimicrobial therapy, remained negative throughout hospitalization, and CT of the head, neck, and chest revealed no alternative source of infection. Despite the absence of microbiological confirmation, the patient exhibited rapid clinical and biochemical improvement, with a marked decline in inflammatory markers, including CRP, PCT, leukocyte count, and neutrophilia, along with gradual improvement of liver and renal function (Figure 3).

**Figure 4:**
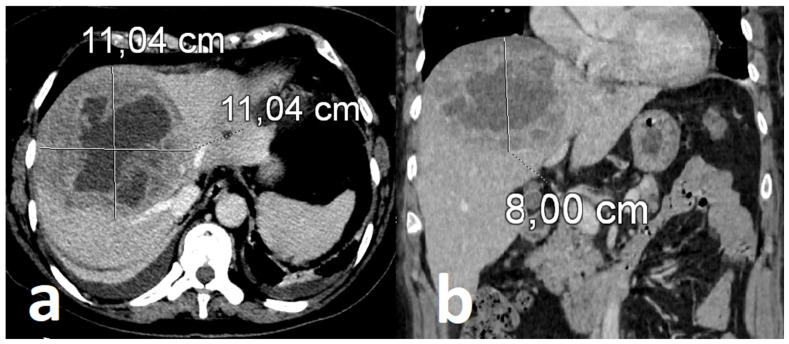
Radiological progression of hepatic abscess measuring approx. 11 × 11 × 8 cm in follow-up CT scan: axial (**a**) and coronal (**b**) plane views.

Follow-up contrast-enhanced CT (serial CT imaging) obtained after three weeks of intravenous antimicrobial therapy revealed continued enlargement of the hepatic lesion despite marked clinical and biochemical improvement (Figure 4). This radiologic-clinical discordance prompted reconsideration of therapeutic strategy and raised concern for insufficient source control.

A CT-guided PCD was subsequently performed one week later, four weeks after the initiation of antimicrobial therapy. The timing of the procedure was influenced by ongoing clinical reassessment and initial patient hesitancy regarding invasive intervention. The procedure was uncomplicated and allowed successful evacuation of purulent material. Cultures obtained from the abscess cavity and drainage system remained negative.

**Figure 5:**
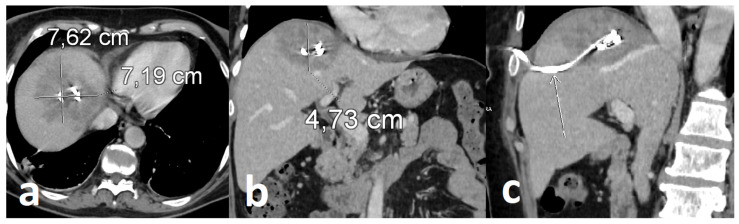
Reduction in size of abscess measuring approx. 7.6 × 7.2 × 4.7 cm after percutaneous catheter drainage procedure (long arrow): axial (**a**), coronal (**b**) and sagittal oblique (**c**) plane views from subsequent contrast-enhanced CT scan.

Follow-up contrast-enhanced CT demonstrated a reduction in lesion size with the drainage catheter appropriately positioned within the abscess cavity, supporting effective source control and early radiological response (Figure 5). Continued antimicrobial therapy was associated with sustained clinical improvement and progressive radiological regression.

After a six-week hospitalization, the patient was discharged in stable condition with the drainage system in situ, without ongoing antibiotic therapy. The catheter was removed two weeks later in the outpatient setting. The total duration of antimicrobial therapy was 34 days. Follow-up imaging over the subsequent twelve months confirmed complete resolution of the hepatic lesion without evidence of recurrence.

## 3. Discussion

Giant CNPLA poses a significant diagnostic and therapeutic challenge, particularly when clinical improvement does not parallel radiological evolution. The present case illustrates how reliance on systemic markers alone may overestimate treatment success and delay adequate source control. In a recent population-based cohort, the 90-day mortality of patients with PLA reached approximately 16%, underscoring the potentially serious course of this infection despite advances in diagnosis and treatment [17].

Severe systemic inflammation and organ dysfunction may occur despite persistently negative microbiological cultures. Culture negativity does not exclude advanced disease and may reflect prior antimicrobial exposure or localized infection inaccessible to routine sampling. Evidence from cohort analyses suggests that patients with CNPLA experience mortality rates and outcomes comparable to microbiologically confirmed disease [10,18], supporting the use of empiric antimicrobial therapy. In the present case, the absence of microbial growth from the abscess cavity may be explained by prolonged broad-spectrum antibiotic therapy prior to drainage, as early antibiotic exposure is known to reduce culture yield.

A 2023 systematic review reported wide variability in antibiotic duration for PLA (pooled mean ~33 days), with mean treatment durations across studies ranging from approximately 8 to 69 days, and highlighted the absence of high-quality evidence defining optimal treatment length, supporting an individualized approach to management based on clinical course and adequacy of source control [19].

The empirical antimicrobial regimen used in this case provided broad-spectrum coverage against both aerobic and anaerobic pathogens. Combination therapy with carbapenem and metronidazole has been reported in clinical practice in the management of PLAs [20]. While meropenem alone exhibits substantial anaerobic activity, the addition of metronidazole may enhance anaerobic coverage; however, it may also represent a degree of therapeutic overlap. In the context of clinical improvement and in the absence of microbiological confirmation of anaerobic infection, early de-escalation of metronidazole could be considered in line with antimicrobial stewardship principles, though optimal strategies for antibiotic simplification remain insufficiently defined [21,22]. Although outpatient management may be appropriate in selected patients, it was not suitable in this case. The need for close clinical monitoring, repeated imaging, and continued intravenous antimicrobial therapy in the setting of evolving disease limited its feasibility.

DM represents one of the most important risk factors for PLA and is the most frequent comorbidity in this population, present in approximately 32–38% of cases [3,23,24]. Increased susceptibility has been observed even among patients achieving recommended glycemic targets [25]. Importantly, DM is not an independent predictor of mortality in PLA [26]. Patients with concomitant DM often require more intensive antimicrobial therapy, including combination regimens and carbapenems [24]. In our patient, well-controlled type 2 DM may still have contributed to the development of a giant, multiloculated abscess and the need for aggressive therapy and timely source control, consistent with evidence that such severe infections may occur even in the absence of overt immunosuppression [27].

In this case, radiological assessment was central to clinical decision-making. CT provides prognostic information, as features such as wall thickness, attenuation, abscess size, and the presence of gas may influence treatment response [28,29]. Serial imaging was crucial in identifying disease progression despite clinical improvement and in guiding timely intervention.

Although antimicrobial therapy is essential, larger (>5 cm) or multiloculated abscesses are more likely to require PCD, particularly in cases with inadequate clinical response, as these features are associated with a higher risk of treatment failure [30,31]. PCD is an effective and less invasive alternative to SD, achieving favorable outcomes in selected patients, [9,30,32], and recent data support its use even in non-liquefied collections [32]. Early intervention facilitates evacuation, reduces bacterial burden, and improves antibiotic effectiveness, whereas delayed drainage may prolong disease course [31,32]. In real-world settings, however, the timing of drainage may be influenced by various clinical and organizational factors.

In the present case, PCD was performed approximately four weeks after initiation of antimicrobial therapy, primarily due to initial patient reluctance, reinforced by early clinical improvement. The observed discrepancy between clinical response and lesion enlargement can be explained by insufficient source control allowing continued accumulation of purulent material, as well as by the natural evolution of abscess formation, with progressive liquefaction from the center toward the periphery, and changes in internal structure. This may result in apparent enlargement on imaging despite initial clinical response, particularly when peripheral portions of the lesion remain in the inflammatory stage and have not yet undergone full cavitation [14]. Following drainage, rapid radiological regression and sustained clinical recovery were observed, while earlier intervention might have limited abscess expansion and shortened hospitalization.

At discharge, the patient demonstrated sustained clinical improvement, normalization of inflammatory markers, and radiological regression following effective drainage. A structured outpatient follow-up was arranged within two weeks, confirming continued clinical stability. The decision to discontinue antimicrobial therapy while maintaining the drainage catheter in situ was based on the assessment of effective source control and stable clinical status.

This case underscores the potential discordance between systemic improvement and local disease control in giant PLA. Clinical stabilization alone may be insufficient to guide management, whereas persistent radiological abnormalities should prompt early reassessment of treatment strategy. The integration of clinical, laboratory, and imaging findings remains essential for optimal management.

## 4. Conclusions

The management of giant PLAs remains complex, particularly in patients with DM, and clinical and microbiological parameters alone may be insufficient to guide treatment decisions. Culture-negative status does not preclude severe disease or the need for aggressive treatment. Early integration of radiological, clinical, and laboratory findings is crucial for timely therapeutic escalation. Image-guided PCD represents an effective and minimally invasive strategy even in large, multiloculated, or poorly liquefied abscesses, and should be considered promptly when radiological progression occurs despite systemic improvement. An individualized, multidisciplinary approach involving close collaboration between internists, radiologists, and interventional specialists remains essential to optimize outcomes in this challenging patient population.

## Data Availability

The data analyzed or generated during the study are available from the corresponding author on reasonable request. The data presented in this study are not publicly available due to patient privacy and ethical restrictions.

## Abbreviations

The following abbreviations are used in this manuscript:

PLA	Pyogenic liver abscess
CNPLA	Culture-negative pyogenic liver abscess
PCD	Percutaneous catheter drainage
DM	Diabetes mellitus
CT	Computed tomography
MRI	Magnetic resonance imaging
CRP	C-reactive protein
PCT	Procalcitonin
AST	Aspartate aminotransferase
ALT	Alanine aminotransferase
GGT	Gamma-glutamyl transferase
HBsAg	Hepatitis B surface antigen
anti-HBc	Hepatitis B core antibody
anti-HBs	Hepatitis B surface antibody
anti-HCV	Hepatitis C virus antibody
HIV	Human immunodeficiency virus
CA-125	Cancer antigen 125
CEA	Carcinoembryonic antigen
AFP	Alpha-fetoprotein
eGFR	Estimated glomerular filtration rate
